# American Indian and Non-Hispanic White Midlife Mortality Is Associated With Medicaid Spending: An Oklahoma Ecological Study (1999–2016)

**DOI:** 10.3389/fpubh.2020.00139

**Published:** 2020-04-29

**Authors:** Mark A. Brandenburg

**Affiliations:** Department of Medicine, Bristow Medical Center, Bristow, OK, United States

**Keywords:** American Indians, Native Americans, non-Hispanic whites, all-cause mortality, Oklahoma Medicaid spending, prescription opioids

## Abstract

**Objective:** A one third reduction of premature deaths from non-communicable diseases by 2030 is a target of the United Nations Sustainable Development Goal for Health. Unlike in other developed nations, premature mortality in the United States (US) is increasing. The state of Oklahoma suffers some of the greatest rates in the US of both all-cause mortality and overdose deaths. Medicaid opioids are associated with overdose death at the patient level, but the impact of this exposure on population all-cause mortality is unknown. The objective of this study was to look for an association between Medicaid spending, as proxy measure for Medicaid opioid exposure, and all-cause mortality rates in the 45–54-year-old American Indian/Alaska Native (AI/AN45-54) and non-Hispanic white (NHW45-54) populations.

**Methods:** All-cause mortality rates were collected from the US Centers for Disease Control & Prevention Wonder Detailed Mortality database. Annual per capita (APC) Medicaid spending, and APC Medicare opioid claims, smoking, obesity, and poverty data were also collected from existing databases. County-level multiple linear regression (MLR) analyses were performed. American Indian mortality misclassification at death is known to be common, and sparse populations are present in certain counties; therefore, the two populations were examined as a combined population (AI/NHW45-54), with results being compared to NHW45-54 alone.

**Results:** State-level simple linear regressions of AI/NHW45-54 mortality and APC Medicaid spending show strong, linear correlations: females, coefficient 0.168, (R^2^ 0.956; *P* < 0.0001; CI95 0.15, 0.19); and males, coefficient 0.139 (R^2^ 0.746; *P* < 0.0001; CI95 0.10, 0.18). County-level regression models reveal that AI/NHW45-54 mortality is strongly associated with APC Medicaid spending, adjusting for Medicare opioid claims, smoking, obesity, and poverty. In females: [R^2^ 0.545; (F)*P* < 0.0001; Medicaid spending coefficient 0.137; *P* < 0.004; 95% CI 0.05, 0.23]. In males: [R^2^ 0.719; (F)*P* < 0.0001; Medicaid spending coefficient 0.330; *P* < 0.001; 95% CI 0.21, 0.45].

**Conclusions:** In Oklahoma, per capita Medicaid spending is a very strong risk factor for all-cause mortality in the combined AI/NHW45-54 population, after controlling for Medicare opioid claims, smoking, obesity, and poverty.

## Introduction

A one third reduction of premature deaths from non-communicable diseases through prevention and treatment in each country by 2030 is a target set forth as part of the United Nations Sustainable Development Goal for Health (1). Unlike in other developed nations, United States (US) premature mortality has been increasing over the last 10 years (2). The most striking reversals in US mortality rates have occurred in middle-aged American Indians (AI/AN) and non-Hispanic whites (NHW) (3, 4). It is imperative that the etiology of this rising premature mortality is identified, in order to reverse this trend.

**Figure 1:**
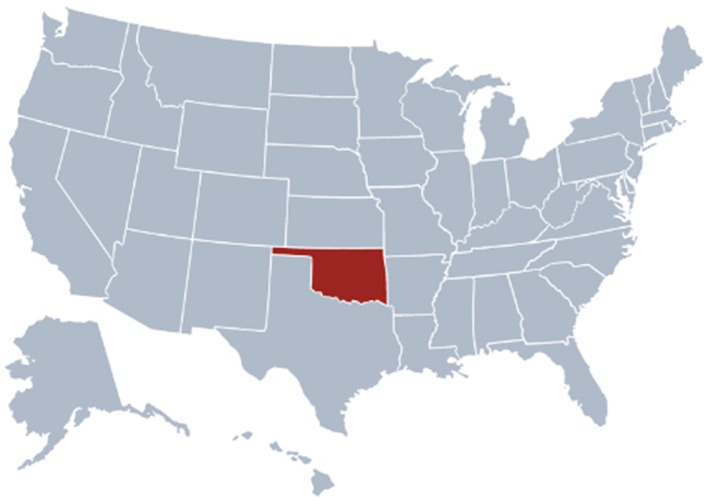
Map showing Oklahoma's geographical location in the USA.

**Figure 2:**
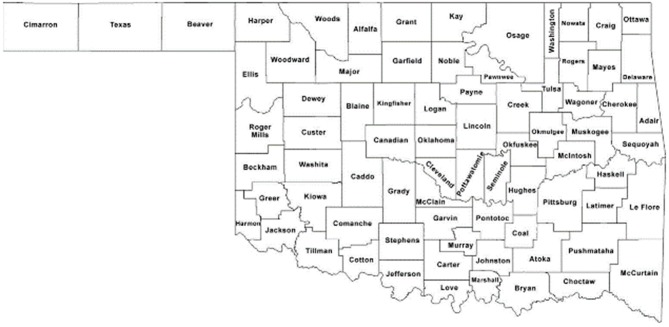
Oklahoma county map.

The midwestern state of Oklahoma (Figure 1) is suffering one of the greatest increases in US all-cause mortality (5). Originally designated Indian Territory in the nineteenth century for the relocation of American Indian tribes forced by the government from their aboriginal lands land, Oklahoma has 77 counties (Figure 2) and a census-reported American Indian/Alaska Native population of ~367,000, representing ~9.3% of Oklahomans (3,943,079) (6).

### American Indian/Alaska Native Mortality

American Indians have been suffering increasing rates of opioid overdose mortality (7), with routine data showing this population to also have the highest unintentional injury, accidental poisoning, and liver disease death rates in the US (8). Due to misclassification of race on death certificates, AI/AN all-cause mortality is believed to be under-estimated by approximately 40%; with most of these misclassifications being as NHW (9). In Washington state the AI/AN population, after correcting for misclassification, had 2.7 times greater incidence of opioid-related overdose deaths compared to whites (10). In Oklahoma, AI/AN and NHW misclassification at the time of death is also well documented, and leads to significant underestimations of AI/AN mortality rates (11). In Oklahoma, temporal patterns of midlife American Indian mortality and Medicaid opioid sales are similar, owing to either common causes of death, misclassification at the time of death, or both (12).

### The US Opioid Epidemic

The United States has been suffering through the historic Opioid Epidemic over the last two decades (13, 14), when healthcare spending has increased substantially (15). The 45-54-year-old age group has been hit hardest by the Opioid Epidemic, and continues to suffer the highest drug- and alcohol-related mortality rates (16). Diversion of prescribed opioids into the broader population is very common (17); hence, it is necessary to study the community impact brought on by these opioid sources.

Oklahoma is consistently among the top 10 states for per capita sales of prescription opioids, and substance abuse-related death (18). From 1994 through 2006, there was a fourfold increase in prescription opioids sales in Oklahoma, when deaths associated with oxycodone, methadone, and hydrocodone increased 12-fold, eightfold, and sevenfold, respectively (19). In Oklahoma, female and male 45-54-year-old populations had the highest rates of unintentional overdose death: 28.8 and 27.7 deaths per 100,000, respectively (20). Wide disparity in opioid prescribing exists across Oklahoma counties (21). The Oklahoma Healthcare Authority conducted its own internal investigation, and identified an association between high-volume opioid pharmacy sales and geographical variation in opioid overdose deaths (22).

Researchers have hypothesized that exposure to Medicaid-funded healthcare is a risk factor for increasing mortality in the 45-54 population due to the over-prescribing of opioids (23, 24). As poverty, smoking, and obesity are well-recognized risk factors for all-cause mortality (25–29), these other known exposures must be included in multivariable regression models when conducting ecological studies that examine Medicaid spending as the primary risk factor for all-cause mortality.

## Objective

The goal of this study was to determine if Medicaid opioid exposure is associated with all-cause mortality at the population level, with the assumption that Medicaid spending is a proxy for population exposure to prescription opioids. The specific objectives of this study are to examine county-level associations between annual per capita (APC) Medicaid spending and all-cause mortality (females and males) in the 45-54-year-old American Indian/Alaska Natives (AI/AN45-54) and non-Hispanic white (NHW45-54) populations; adjusting for APC Medicare opioid claims, and the prevalence of smoking, obesity, and poverty.

## Methods

### Mortality Data

All-cause mortality data from the CDC Wonder Detailed Mortality database were restricted to male or female AI/AN45-54 and NHW45-54 groups (30); with the International Classification of Diseases (ICD-10) codes published by the World Health Organization utilized for cause-specific mortality classifications. Misclassification between AI/AN and NHW populations and sparse AI/AN data at the county-level were managed by combining the data of these two populations: AI/AN45-54 and NHW45-54 (AI/NHW45-54).

**Figure 3:**
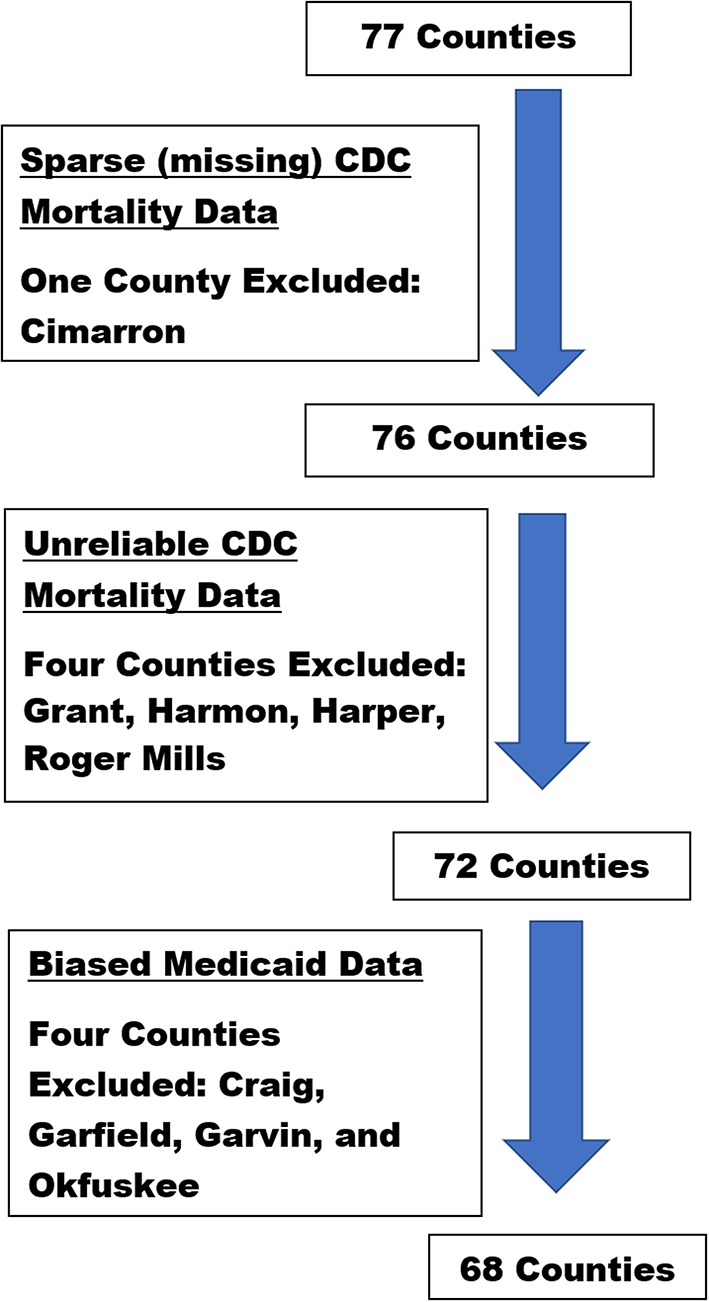
Oklahoma county-level female dataset selection process and rationale.

**Figure 4:**
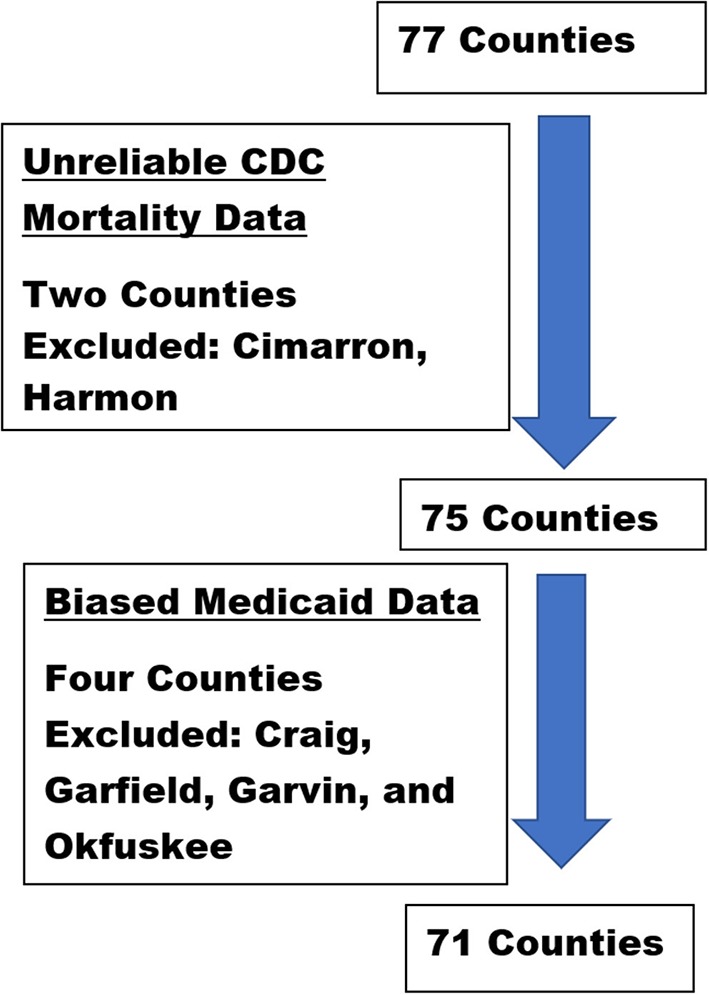
Oklahoma county-level male dataset selection process and rationale.

Annual data for each county were aggregated across a span of 17 years (1999–2016). The aggregated female AI/NHW 45-54 all-cause mortality data from one rural county (e.g., Cimarron) was suppressed by the CDC due to sparse data; and mortality data from four additional rural counties (e.g., Grant, Harmon, Harper, and Roger Mills) were sparse enough to be deemed unreliable. Female all-cause mortality data from these five counties were excluded from data analyses (Figure 3). The aggregated male AI/NHW45-54 all-cause mortality data from two rural counties (e.g., Cimarron and Harmon), were unreliable; data from these two counties were excluded from analyses (Figure 4).

### Medicaid Data

The annual (2000–16) Oklahoma Healthcare Authority reports with state and county enrollee and spending data are published online. Data on county-level, annual per capita Medicaid spending were aggregated as means, and used as the primary exposure variable in the MLR analyses. Four additional sets of county data (e.g., Craig, Garfield, Garvin, and Okfuskee) were excluded from analyses because the Oklahoma Healthcare Authority identified them as biased, due to existing inpatient facilities to which disproportionate per capita shares of Medicaid funding are allotted.

### Co-variate Data

**Figure 5:**
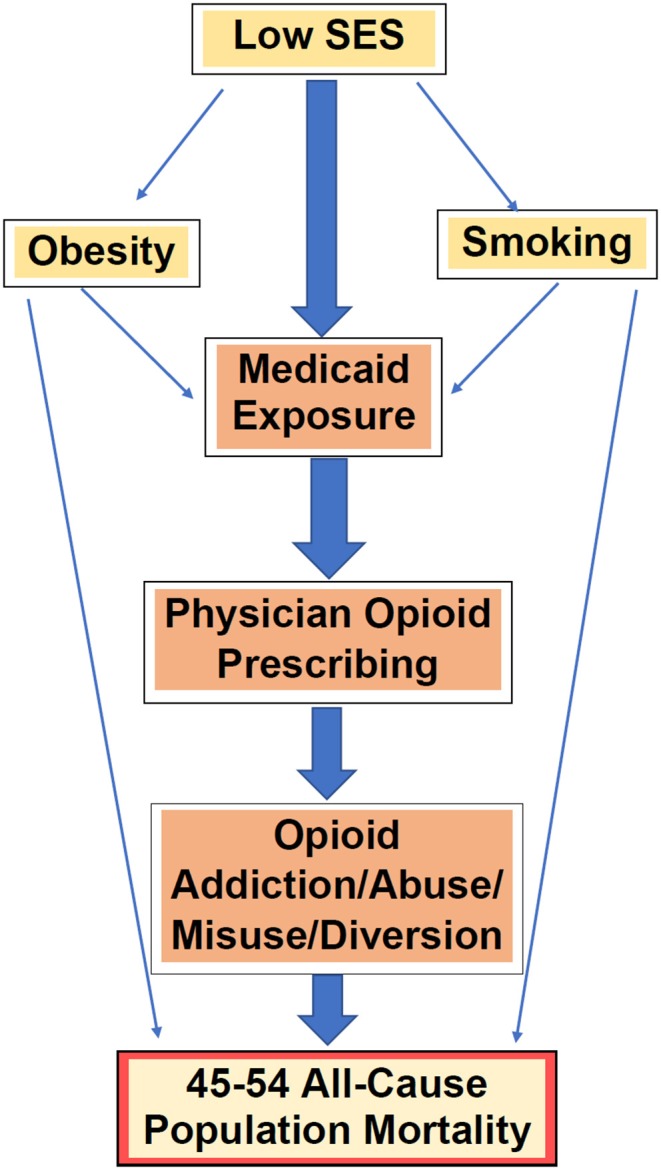
A priori confounders & effect modifiers in the paradigm of Medicaid exposure (as proxy for prescription opioids) and all-cause mortality.

The co-variates chosen for MLR analyses each have a priori evidence for association with Medicaid, independent risk for mortality, while not being on the conceptual pathway of risk between Medicaid and mortality (Figure 5). These associations were confirmed with the data collected in this study, and are included in Supplementary Tables 3,4.

Medicare Part D opioid claims data per Oklahoma county were made available online, by Centers for Medicare & Medicaid Services, for 2013 (31) and 2014 (32). For each county, the mean annual claims per capita was derived from these data. Co-variate data on smoking, obesity, and poverty were independently obtained through population surveys. Survey data on smoking and obesity were obtained from University of Wisconsin/Robert Wood Johnson Foundation County Health Rankings and Roadmaps database (33). Smoking was defined as currently smoking (2016–2018) (34), and obesity was defined as body mass index >30 (2010–2018) (35). The US Census Bureau county-level data on poverty are available online for 2005–2015 (36). The mean annual proportion of county populations for each co-variate was calculated for each county from these datasets (Supplementary Table 3d). Histograms allowed for the visualization of relatively normal distributions (Supplementary Figures 1–4).

Statistical analyses were performed using Stata 15.1 software. At the state-level, simple linear regression analyses were performed on AI/AN45-54, NHW45-54, AI/NHW45-54 mortality, each against APC Medicaid spending. At the county-level, simple linear regression models were constructed to assess crude associations between AI/NHW 45–54 mortality (by gender) and mean APC Medicaid spending. Additional explanatory co-variates were added to MLR models, to adjust for confounding.

### Data Handling

All data were anonymized by the database owners prior to the commencement of this project. Mortality data were not used in the final models if suppressed by CDC, due to sparseness. Annual county mortality data with fewer than ten deaths (0–9) were suppressed.

### Tested Assumptions of Linear Regression

All variables were plotted on histograms (assumption of normality). The crude relationships between mortality and Medicaid were examined by scatterplot (assumption of linearity). Quadratic and cubic functions of each predictor variable were also added to the final models in search of non-linearity. Scatterplots and Breusch–Pagan tests were carried out to assess residual mortality distributions across the fitted line and levels of the exposure variable (assumption of homoscedasticity). Variance inflation factors were calculated in the multiple regression models to assess for multicollinearity.

## Results

### Statewide

**Figure 6:**
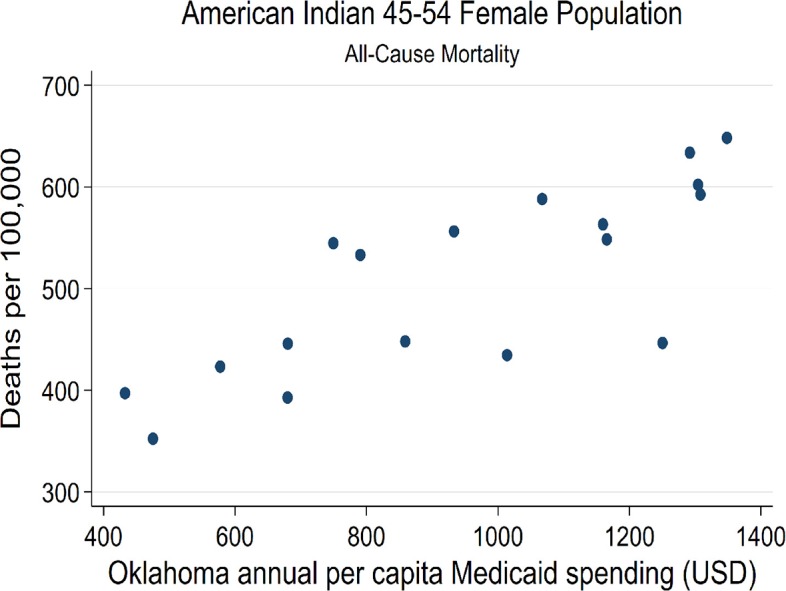
State-level Oklahoma Medicaid spending linear regression plot (crude association) with female AI45-54 all-cause mortality.

**Figure 7:**
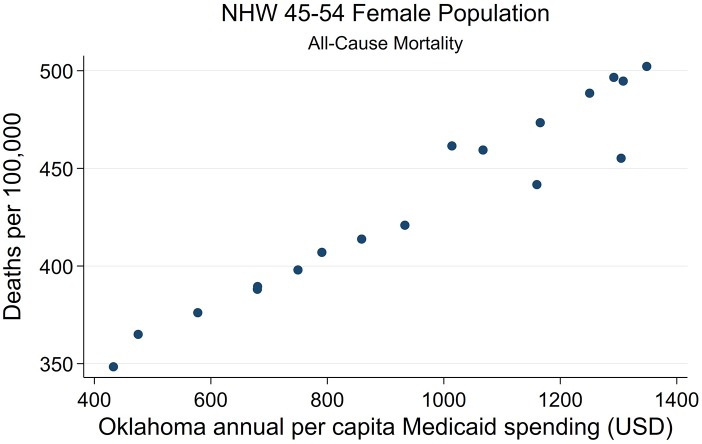
State-level Oklahoma Medicaid spending linear regression plot (crude association) with female NHW45-54 all-cause mortality.

**Figure 8:**
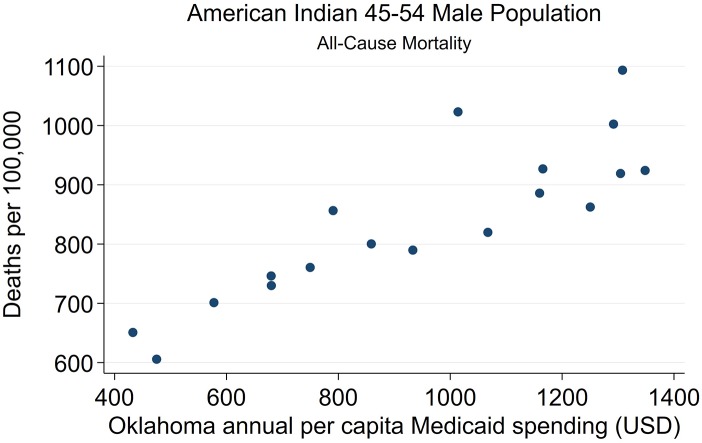
State-level Oklahoma Medicaid spending linear regression plot (crude association) with male AI45-54 all-cause mortality.

**Figure 9:**
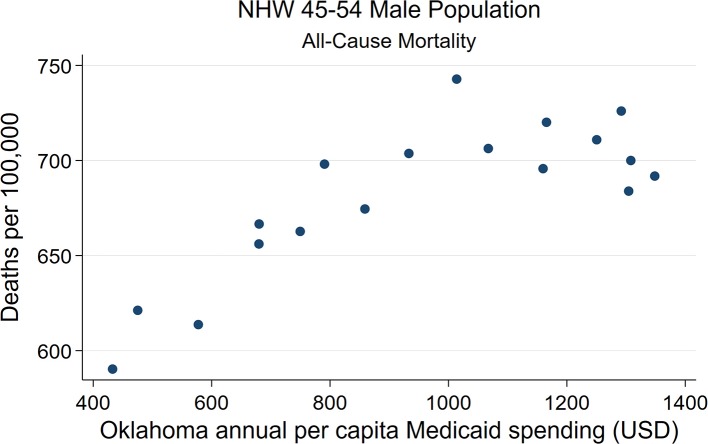
State-level Oklahoma Medicaid spending linear regression plot (crude association) with male NHW45-54 all-cause mortality.

Strong associations at the state-level were found between Medicaid spending and AI/NHW45-54 population all-cause mortality, in males and females. Simple linear regressions of AI/NHW45-54 mortality and APC Medicaid spending show strong correlations: in females, coefficient 0.168, (R^2^ 0.956; *P* < 0.0001; CI95 0.15, 0.19); and in males, coefficient 0.139 (R^2^ 0.746; *P* < 0.0001; CI95 0.10, 0.18) Supplementary Tables 2a–c, Figures 6–9).

### County-Level

County-level regression models reveal that AI/NHW45-54 mortality is strongly associated with mean APC Medicaid spending, after adjusting for Medicare opioid claims, smoking, obesity, and poverty. In females: [R^2^ 0.545; (F)*P* < 0.0001; Medicaid spending coefficient 0.137; *P* < 0.004; 95% CI 0.05, 0.23]. In males: [R^2^ 0.719; (F)*P* < 0.0001; Medicaid spending coefficient 0.330; *P* < 0.001; 95% CI 0.21, 0.45]. For every $100 in per capita spending by Medicaid, there were an additional 14 female and 33 male AI/NHW45-54 deaths per 100,000. The second strongest risk factor for association with population all-cause mortality in males and females was Medicare opioid claims (Tables 1,2).

**TABLE 1a:** Multiple linear regression analyses results AI/NHW 45-54 FEMALE all-cause mortality and mean APC Medicaid spending.

**68 counties**	**Unadjusted**	**Model 1**	**Model 2**	**Model 3**	**Model 4**	**Model 5**
R2	0.484	0.527	0.539	0.540	0.529	0.545
Adjusted R2	0.476	0.513	0.517	0.519	0.507	0.509
F (df)	61.92 (1, 66)	36.32 (2, 65)	24.90 (3, 64)	25.07 (3, 64)	24.00 (3, 64)	14.88 (3, 62)
*P* (F)	<0.0001	<0.0001	<0.0001	<0.0001	<0.0001	<0.0001
Medicaid β	0.214	0.180	0.144	0.157	0.167	0.137
Medicaid Wald *P*	<0.001	<0.001	0.001	<0.001	<0.001	0.004
CI95	0.160 0.269	0.120 0.239	0.060 0.227	0.088 0.226	0.086 0.248	0.046 0.227
VIF*	1.0	1.29 1.29	2.55 2.34 1.29	1.75 1.38 1.32	2.34 2.08 1.29	4.19 3.34 2.94 1.61 1.34
Breusch-Pagan chi2 P**	0.15 0.70	0.590.442	0.00 0.9536	0.15 0.6970	0.42 0.516	0.000 0.954
Medicare Opioid Claims Wald *P*		0.017	0.020	0.010	0.018	0.015
Smoking Wald *P*			0.226			0.479
Obesity Wald *P*				0.192		0.360
Poverty Wald *P*					0.634	0.932

* *Variance Inflation Factors (checking for multicollinearity)*.

** *Breusch-Pagan/Cook-Weisberg Tests (checking for linear heteroskedasticity) (37)*.

*Model 1–adjusted for Medicare opioid claims*.

*Model 2–adjusted for Medicare opioid claims & smoking*.

*Model 3–adjusted for Medicare opioid claims & obesity*.

*Model 4–adjusted for Medicare opioid claims & poverty*.

*Model 5–adjusted for Medicare opioid claims, smoking, obesity & poverty*.

**TABLE 1b:** Multiple linear regression analyses results AI/NHW 45-54 MALE all-cause mortality and mean APC Medicaid spending.

**71 counties**	**Unadjusted**	**Model 1**	**Model 2**	**Model 3**	**Model 4**	**Model 5**
R2	0.694	0.703	0.714	0.709	0.712	0.719
Adjusted R2	0.689	0.694	0.701	0.696	0.699	0.698
F (df)	156.29 (1, 69)	80.21 (2, 68)	55.77 (3, 67)	54.29 (3, 67)	55.26 (3, 67)	33.32 (5, 65)
*P* (F)	<0.0001	<0.0001	<0.0001	<0.0001	<0.0001	<0.0001
Medicaid β	0.448	0.419	0.354	0.390	0.365	0.330
Medicaid Wald *P*	<0.001	<0.001	<0.001	<0.001	<0.001	<0.001
CI95	0.376 0.519	0.338 0.501	0.242 0.467	0.295 0.485	0.257 0.473	0.208 0.451
VIF*	1.000	1.33 1.33	2.57 2.37 1.34	1.80 1.37 1.36	2.36 2.10 1.33	4.30 3.47 2.98 1.58 1.40
Breusch-Pagan chi2 P**	1.68 0.195	1.50 0.221	0.89 0.347	0.90 0.343	0.67 0.412	0.28 0.597
Medicare Opioid Claims Wald *P*		0.166	0.210	0.117	0.188	0.161
Smoking Wald *P*			0.102			0.594
Obesity Wald *P*				0.237		0.373
Poverty Wald *P*					0.135	0.414

* *Variance Inflation Factors (checking for multicollinearity)*.

** *Breusch-Pagan/Cook-Weisberg Tests (checking for linear heteroskedasticity) (37)*.

*Model 1–adjusted for Medicare opioid claims*.

*Model 2–adjusted for Medicare opioid claims & smoking*.

*Model 3–adjusted for Medicare opioid claims & obesity*.

*Model 4–adjusted for Medicare opioid claims & poverty*.

*Model 5–adjusted for Medicare opioid claims, smoking, obesity & poverty*.

**TABLE 2a:** Multiple linear regression analyses results NHW 45-54 FEMALE all-cause mortality and mean APC Medicaid spending.

**66 counties**	**Unadjusted**	**Model 1**	**Model 2**	**Model 3**	**Model 4**	**Model 5**
R2	0.502	0.558	0.558	0.558	0.560	0.563
Adjusted R2	0.494	0.544	0.537	0.519	0.539	0.527
F (df)	64.56 (1, 64)	39.72 (2, 63)	26.06 (3, 62)	26.12 (3, 62)	26.31 (3, 62)	15.46 (5, 60)
*P* (F)	<0.0001	<0.0001	<0.0001	<0.0001	<0.0001	<0.0001
Medicaid β	0.234	0.194	0.194	0.189	0.178	0.177
Medicaid Wald *P*	<0.001	<0.001	0.001	<0.001	<0.001	<0.001
CI95	0.176, 0.292	0.132, 0.256	0.107, 0.280	0.114, 0.263	0.094, 0.262	0.081, 0.273
VIF*	1.0	1.26, 1.26	2.42, 1.26, 2.24	1.79, 1.28, 1.45	2.29, 1.26, 2.07	4.22, 3.46, 2.91, 1.76 1.30
Breusch-Pagan chi2, P**	3.99 0.046	2.83 0.093	2.85 0.091	2.96 0.085	3.59 0.058	3.52 0.061
Medicare Opioid Claims Wald *P*		0.007	0.007	0.007	0.007	0.007
Smoking Wald *P*			0.979			0.576
Obesity Wald *P*				0.779		0.629
Poverty Wald *P*					0.563	0.428

* *Variance Inflation Factors (checking for multicollinearity)*.

** *Breusch-Pagan/Cook-Weisberg Tests (checking for linear heteroskedasticity) (37)*.

*Model 1–adjusted for Medicare opioid claims*.

*Model 2–adjusted for Medicare opioid claims & smoking*.

*Model 3–adjusted for Medicare opioid claims & obesity*.

*Model 4–adjusted for Medicare opioid claims & poverty*.

*Model 5–adjusted for Medicare opioid claims, smoking, obesity & poverty*.

**TABLE 2b:** Multiple linear regression analyses results NHW 45-54 MALE all-cause mortality and mean APC Medicaid spending.

**71 counties**	**Unadjusted**	**Model 1**	**Model 2**	**Model 3**	**Model 4**	**Model 5**
R2	0.707	0.737	0.741	0.739	0.751	0.754
Adjusted R2	0.702	0.730	0.730	0.728	0.740	0.735
F (df)	166.16 (1, 69)	95.39 (2, 68)	63.80 (3, 67)	63.28 (3, 67)	67.45 (3, 67)	39.87 (5, 65)
*P* (F)	<0.0001	<0.0001	<0.0001	<0.0001	<0.0001	<0.0001
Medicaid β	0.430	0.379	0.345	0.364	0.317	0.310
Medicaid Wald *P*	<0.001	<0.001	<0.001	<0.001	<0.001	<0.001
CI95	0.363 0.496	0.306 0.452	0.243 0.447	0.278 0.449	0.222 0.413	0.202 0.419
VIF*	1.000	1.33 1.33	2.57 2.37 1.34	1.80 1.37 1.36	2.36 2.10 1.33	4.30 3.47 2.98 1.58 1.40
Breusch-Pagan chi2 P**	0.97 0.3240	2.57 0.109	2.07 0.150	1.94 0.163	1.05 0.305	0.57 0.4505
Medicare Opioid Claims Wald *P*		0.006	0.008	0.005	0.007	0.006
Smoking Wald *P*			0.347			0.590
Obesity Wald *P*				0.486		0.412
Poverty Wald *P*					0.056	0.072

* *Variance Inflation Factors (checking for multicollinearity)*.

** *Breusch-Pagan/Cook-Weisberg Tests (checking for linear heteroskedasticity) (37)*.

*Model 1–adjusted for Medicare opioid claims*.

*Model 2–adjusted for Medicare opioid claims & smoking*.

*Model 3–adjusted for Medicare opioid claims & obesity*.

*Model 4–adjusted for Medicare opioid claims & poverty*.

*Model 5–adjusted for Medicare opioid claims, smoking, obesity & poverty*.

### Multiple Linear Regression Assumptions

#### Linearity

**Figure 10:**
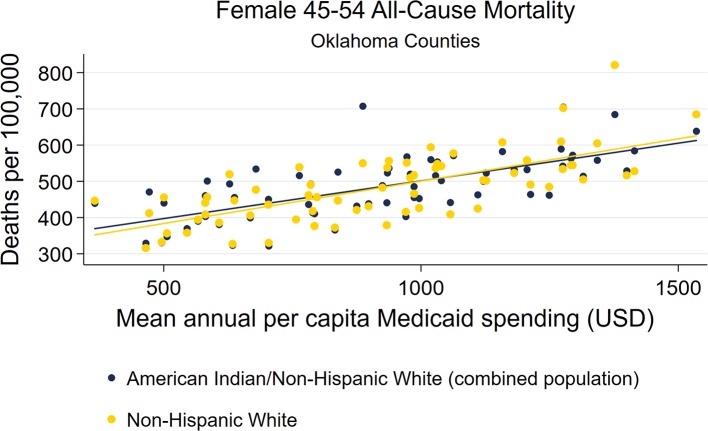
County-level Oklahoma Medicaid spending linear regression plot (crude association) with female AI/NHW45-54.

**Figure 11:**
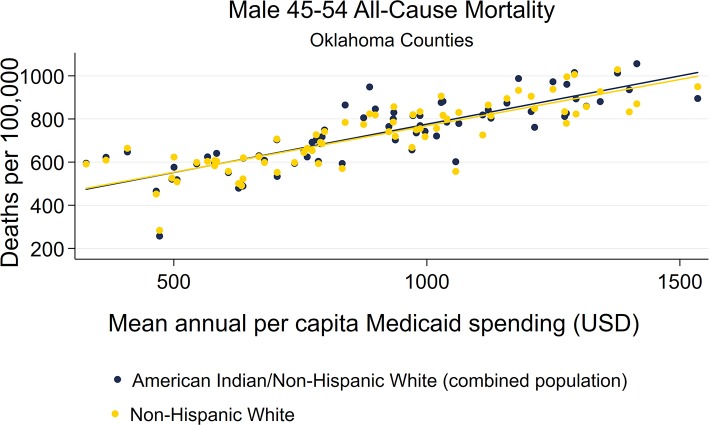
County-level Oklahoma Medicaid spending linear regression plot (crude association) with male AI/NHW45-54.

Scatterplots of female and male AI/NHW45-54 mortality and mean Medicaid spending (2000-2016) both demonstrated linear relationships, and were very similar to those of NHW45-54 (Figures 10–11).

#### Multivariate Normality

Mortality, Medicaid spending, exposure variables, and co-variate data sets all had approximately normal distributions (Supplementary Figures 1–4, Supplementary Table 3a-d). Primary exposure and outcome variables were examined for non-variability of predictor- and fitted-versus residual distributions by scatterplots, and all distributions were all found to be randomly distributed (Supplementary Figures 5a,b).

#### Multicollinearity and Heteroscedasticity

In all MLR models, including the final, adjusted model with all co-variates, correlations between mean APC Medicaid spending and mortality were very strong (*P* < 0.0001; Medicaid Wald *P* < 0.001) (Tables 1, 2). Comparisons of the AI/NHW45-54 MLR analyses results with those obtained using the NHW45-54 datasets found the model outputs to be very similar.

In the female dataset, the presence of multicollinearity and heteroskedasticity both appear in the MLR model that includes all co-variates: mean APC Medicare opioid claims, smoking, obesity, and poverty; whereas neither is present in the models with either poverty or smoking separately. The strongest MLR model demonstrating the association between AI/NHW45-54 population all-cause mortality and mean APC Medicaid spending included the co-variates of mean APC Medicare opioid claims and poverty: [R^2^ 0.529; (F)*P* < 0.0001; Medicaid spending coefficient 0.167; *P* < 0.001; 95% confidence interval (CI95) 0.09, 0.25]. Medicaid spending and Medicare opioid claims were consistently associated with mortality; while poverty was not associated with mortality in the models.

In the male dataset, multicollinearity was seen in the MLR model that includes all co-variates: mean APC Medicare opioid claims, smoking, obesity, and poverty; but was not seen in the models with either poverty or smoking separately. The strongest MLR model demonstrating the association between AI/NHW45-54 all-cause mortality and mean APC Medicaid spending included the following co-variates of mean APC Medicare opioid claims and poverty: [R^2^ 0.712; (F)*P* < 0.0001; Medicaid spending coefficient 0.365; *P* < 0.001; CI95 0.26, 0.47, respectively. Only Medicaid spending was associated with mortality in the models.

### Confounder Assumptions

All predictor variables showed statistically significant, positive correlations with AI/NHW45-54 population all-cause mortality (deaths per 100,000). The strongest correlations with mortality were those seen with mean Medicaid spending, in which the Pearson R correlation coefficients for females and males were 0.696 and 0.833, respectively (Supplementary Table 4a–b). All confounding variables showed statistically significant, positive correlations with mean Medicaid Spending.

### Post-Estimation Analyses

Using quadratic functions of each predictor variable in the final model, Medicaid spending, Medicare opioid claims, and poverty, only the male AI/NHW45-54 regression model showed evidence of non-linearity; while the Medicaid spending *p* < 0.001, the quadratic functions of Medicare opioid claims had a *P* = 0.034, and of poverty had a *P* = 0.005. With these additional variables included in the male dataset, a strengthening of the model was seen: [R^2^ 0.752; (F)*P* < 0.0001; Medicaid spending coefficient 0.351; *P* < 0.001; CI 0.25, 0.45], as compared to the model without the quadratic functions of Medicare opioid claims and poverty [R^2^ 0.712; (F)*P* < 0.0001; Medicaid spending coefficient 0.365; *P* < 0.001; CI95 0.26, 0.47].

Mixed effect regression of NHW45-54 mortality and mean APC Medicaid spending, adjusted for all co-variates together, were very consistent with the corresponding MLR models: female mortality dataset Medicaid spending coefficient 0.137 (*P* < 0.0001; CI95 0.07 0.23); male mortality dataset Medicaid spending coefficient 0.330 (*P* < 0.0001; CI95 0.22, 0.44).

## Discussion

Medicaid spending is the strongest predictor for AI/NHW45-54 population all-cause mortality in Oklahoma. Medicare opioid claims is the next strongest predictor. In the female population, none of the covariates smoking, obesity, and poverty, are significant risk factors when adjusted for Medicaid spending and Medicare opioid claims. In the male population, only Medicaid spending, Medicare opioid claims, and poverty are risk factors in the models, with Medicaid spending the strongest exposure variable associated with mortality.

Consistency was found between APC Medicaid spending and APC Medicare opioid claims in association with mortality. Medicaid spending is targeted to a different population of patients (i.e., low income persons) than Medicare (i.e., >65 years, or disabled). That both healthcare exposure variables are strongly associated with AI/NHW45-54 mortality, brings into great focus the common exposure of physician-prescribed opioids.

These findings are important, as this is the first study to look for an association between the exposure of prescription opioids and all-cause midlife mortality—and more specific, being adjusted for poverty, smoking, and obesity. And indeed, a very strong, positive association between Medicaid and all-cause midlife mortality was found. The association between Medicaid and mortality is stronger than the association of any other of the well-recognized risk factors: poverty, smoking, and obesity. The mere idea that a healthcare system presents a risk factor for population mortality is novel. Whether this association between Medicaid spending and premature midlife mortality will be found in other states is not predictable; similar studies looking at Medicaid as a population mortality risk factor must now be conducted before this can be ruled out.

### Prescription Opioids and Population All-Cause Mortality

While opioids are associated with overdose death, they are also associated with a variety of medical conditions that can have fatal outcomes. Prescription opioid use was recently found to be a risk factor for all-cause morbidity and mortality in hospitalized patients (38). Various medical co-morbidities are known to be associated with opioid abuse and addiction: infections (e.g., endocarditis, osteomyelitis, septic arthritis, or epidural abscess) (39), immune compromise (40), and sleep-disordered breathing (41). Preoperative prescription opioid abuse and dependence is associated with in-hospital, all-cause mortality after orthopedic surgery (42).

At the population level, one recent study found that an expanded pharmaceutical industry was associated with increasing female all-cause mortality rates (43). Recent time series studies of six states in the US Deep South found that prescription opioid sales correlate strongly with medical-cause mortality (44), and even stronger with female cardiovascular mortality (45). In both of the aforementioned two studies, medical mortality rates lagged behind prescription opioid sales by approximately one year—temporality being a necessary component to a causal model for future investigations. Importantly, the mortality data in these studies excluded overdoses, suicides, homicides, and traumatic cause of death. Etiologies for these increasing medical death rates are not known. Nonetheless, when looking at the impact of the Opioid Epidemic at the population level, an examination of all-cause morbidity and mortality is necessary, rather than simply examining overdose death data.

### Medicaid Opioids

Created in 1965, Medicaid is a state and federal health insurance program for low income citizens. Public health experts at the Washington State Department of Health and US Centers for Disease Control & Prevention (CDC) have expressed concern about the high prevalence of opioid-prescribing to Medicaid patients (46).

In a study of 1,772,632 opioid prescriptions for Medicaid patients, one quarter of patients had at least one indicator of potential misuse (47). In Tennessee, the risk of all-cause mortality in Medicaid patients prescribed long-acting opioids was 1.6 times greater than the cohort prescribed other prescribed anticonvulsants or cyclic antidepressants (48). In 2015, an estimated 30% of Americans with opioid addiction received healthcare from Medicaid programs (49). The state of West Virginia Department of Health and Human Resources reported that 71% overdose fatalities had Medicaid exposure in the 12 months prior to their death (50). In Washington state, the risk of opioid-overdose related death for a Medicaid enrollee was 5.7 times the risk for a person not enrolled in Medicaid (51). In a national case-control study using the Medicaid Analytic eXtract database of 37.5 million patients, a strong association was found between Medicaid spending and prevalence of opioid abuse and dependence in Medicaid patients (52).

### Confounding

Smoking, obesity, and poverty each confound the association between Medicaid spending and AI/NHW45-54 mortality, with poverty having the strongest attenuating effect. Poverty in Oklahoma has been slowly rising. The US Census Bureau shows the Oklahoma poverty rate to have been 14.7% in 1999, 16.2% in 2010, and 16.5 in 2016 (53). The question is whether poverty is more an upstream determinant of Medicaid opioids on the pathway toward Oklahoma mortality, than a risk factor itself for mortality. Could another yet un-discovered, un-measured, downstream variable be confounding the Medicaid-Mortality association? Possibly, but until such a risk factor is discovered Medicaid prescription opioids should garner the attention of US epidemiologists, public health entities, economists, and sociologists, as being the most important risk factor for rising US midlife mortality.

### Assumptions With Data Collected

An assumption was made that the aggregated, annual data points for Oklahoma counties were representative of the 2000–16 timeframe. Strong, crude associations between mortality were found, giving credibility to this assumption. Additional assumptions for the final MLR models include validity of the county-level measurements of prevalence from surveys of smoking and obesity, as these data points are estimates of the true population prevalence of each. Assumptions unable to be tested, are that within counties constant and regression coefficient/slope coefficients are homogenous; and that random errors within data sets are uncorrelated with each other.

### Limitations

This is an ecological study, and as such causal conclusions or assumptions about individual or community-level AI/NHW 45-54 mortality risk factors are not valid. Medicaid spending could be a proxy measure for overall prescription opioid spending, as pharmaceutical market forces extend beyond Medicaid prescribers. Data from the Veterans Administration, Indian Health Service, and Medicare or private insurance were not available to the extent that they could be applied to regression models. These conclusions are not generalizable across the US; the effect of Medicaid exposure on all-cause AI/NHW45-54 mortality might be very different in other states. Nonetheless, the US remains in the throes of both the Opioid Epidemic that has claimed the lives of many hundreds of thousands of Americans and a simultaneous decrease in life expectancy—the primary risk factors for both crises should be sought out with due diligence in every state, so the trend of rising premature mortality can be reversed. If Medicaid opioid prescribing is the common risk factor for both crises, then it is especially critical to examine and correct.

### Future Directions

Detailed study of Medicaid data at the individual level would provide valuable insight into increasing AI/AN45-54 mortality. Specifically, data on opioid prescribing rates, medical examiner reports, and geographical locations of the deceased would allow for case-control and cohort studies that could better characterize the effects on AI/AN45-54 mortality. Prescribing data from the Oklahoma Health Care Authority, Indian Health Service, Oklahoma Bureau of Narcotics and Dangerous Drugs, and Medical Examiner's Office, would allow closer examination of physicians associated with geographic hotspots of overprescribing and mortality. Medicaid resource utilization, and opioid prescribing, should be scrutinized in every state to determine if associations exist between Medicaid spending and mortality.

### The Opioid Epidemic: a Global Threat?

While the Opioid Epidemic has not yet gone global, increases in opioid prescribing outside the US have been reported in Canada (54), Serbia (55), the United Kingdom (56), Germany (57), and in several other European countries (58). Future studies will need to be conducted as global trends are monitored, and the impact of prescription opioids on population morbidity and mortality measured. In order to achieve a reduction of premature deaths, as set forth in the United Nations Sustainable Development Goal for Health, preventing excessive opioid prescribing by healthcare systems will be important, but critical if this exposure is linked to all-cause mortality increases.

## Conclusion

In Oklahoma, per capita Medicaid spending is a very strong risk factor for all-cause mortality in the combined populations of American Indian/Alaska Natives (AI/AN) and non-Hispanic whites (45–54-years-old), both males and females, after controlling for Medicare opioid claims, smoking, obesity, and poverty.

## Data Availability Statement

The datasets generated for this study will not be made publicly available They are readily available online, and the links are provided in the paper.

## Author Contributions

The author conceived of this research, performed the background work, collected the data, performed all statistical analyses, and wrote the paper. This research was originally conducted as an EPM500 Project Report, as required during the author's Epidemiology Master's degree studies at the London School of Hygiene and Tropical Medicine (UK).

## Conflict of Interest

The authors declare that the research was conducted in the absence of any commercial or financial relationships that could be construed as a potential conflict of interest.

## Supplementary Material

Supplementary Figure 1a Histogram—Mean Annual County Medicare Opioids–Female Dataset.

Supplementary Figure 1b Histogram—Mean Annual County Medicare Opioids–Male Dataset.

Supplementary Figure 2a Histogram—County Population Obesity–Female Dataset.

Supplementary Figure 2b Histogram—County Population Obesity–Male Dataset.

Supplementary Figure 3a County Smoking–Female Dataset.

Supplementary Figure 3b County Smoking–Male Dataset.

Supplementary Figure 4a Histogram—County Population in Poverty–Female Dataset.

Supplementary Figure 4b Histogram—County Population in Poverty–Male Dataset.

Supplementary Figure 5a Residuals vs. Fitted MAPC Medicaid vs. NHW45-54–Female Dataset.

Supplementary Figure 5b Residuals vs. Fitted MAPC Medicaid vs. NHW45-54–Male Dataset.

Supplementary Table 1 Online, publicly accessible databases used in this study.

Supplementary Table 2a Simple linear regressions between annual Oklahoma AI-NHW45-54 all-cause mortality and Medicaid spending per capita statewide (1999-16).

Supplementary Table 2b Simple linear regressions between annual Oklahoma AI45-54 all-cause mortality and Medicaid spending per capita statewide (1999-16).

Supplementary Table 2c Simple linear regressions between annual Oklahoma NHW45-54 all-cause mortality and Medicaid spending per capita statewide (1999-16).

Supplementary Table 3 County-Level Variable Summaries.

Supplementary Table 3a Primary Outcome: NHW-45-54 All-Cause Mortality^*^ (2000-16). ^*^Deaths per 100 000 persons .

Supplementary Table 3b Primary Exposure: MAPC Medicaid Spending^*^ (2000-16). ^*^U.S. Dollars.

Supplementary Table 3c Secondary Exposure: MAPC Medicare Opioid Claims (2013-14).

Supplementary Table 3d Covariate Exposure^*^ Variables. ^*^Proportion of Individuals Surveyed.

Supplementary Table 4 Correlations between mortality and explanatory variables.

Supplementary Table 4a Pearson r correlations between county level AI-NHW45-54 mortality and explanatory variables (FEMALE mortality dataset).

Supplementary Table 4b Pearson r correlations between county level AI-NHW45-54 mortality and explanatory variables (MALE mortality dataset).
