# Supplementary figures and images for: American Indian and Non-Hispanic White Midlife Mortality Is Associated With Medicaid Spending: An Oklahoma Ecological Study (1999–2016)
Source: Front Public Health. 2020 Apr 29;8:139. doi: 10.3389/fpubh.2020.00139 (PMC7202289; doi:10.3389/fpubh.2020.00139)

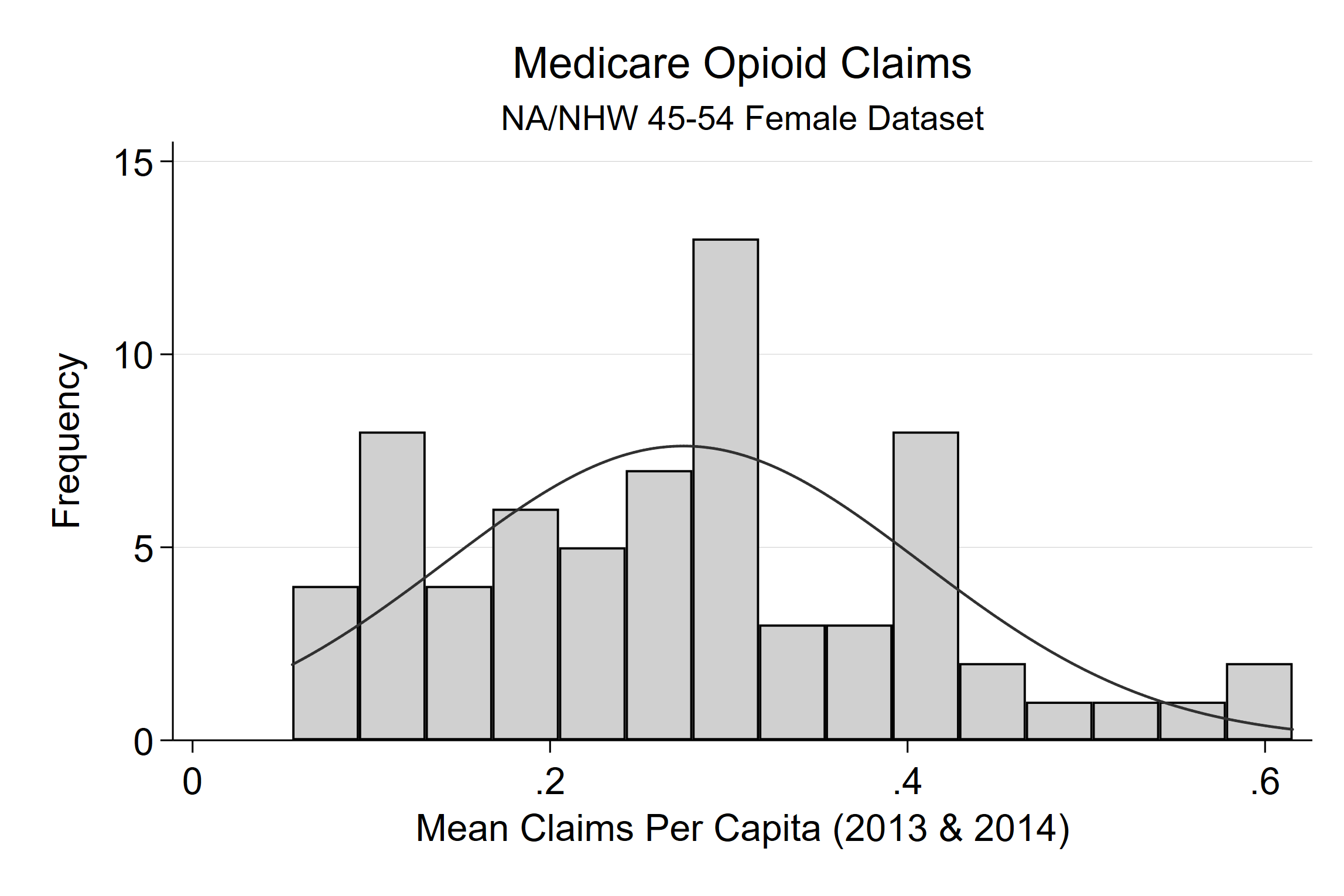

Supplement: Supplementary Figure 1a — Histogram—Mean Annual County Medicare Opioids–Female Dataset. [file Image_1.PNG]

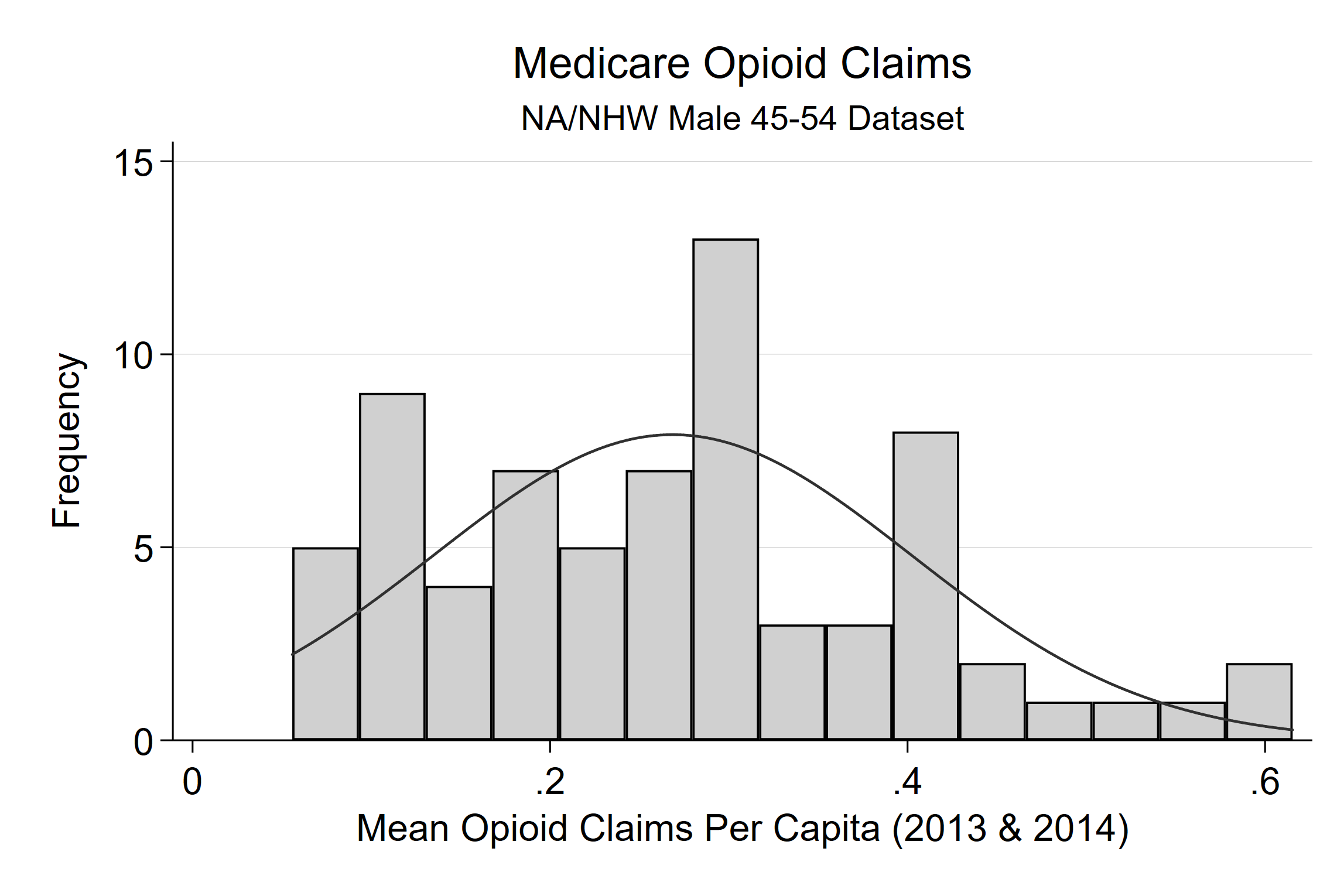

Supplement: Supplementary Figure 1b — Histogram—Mean Annual County Medicare Opioids–Male Dataset. [file Image_2.PNG]

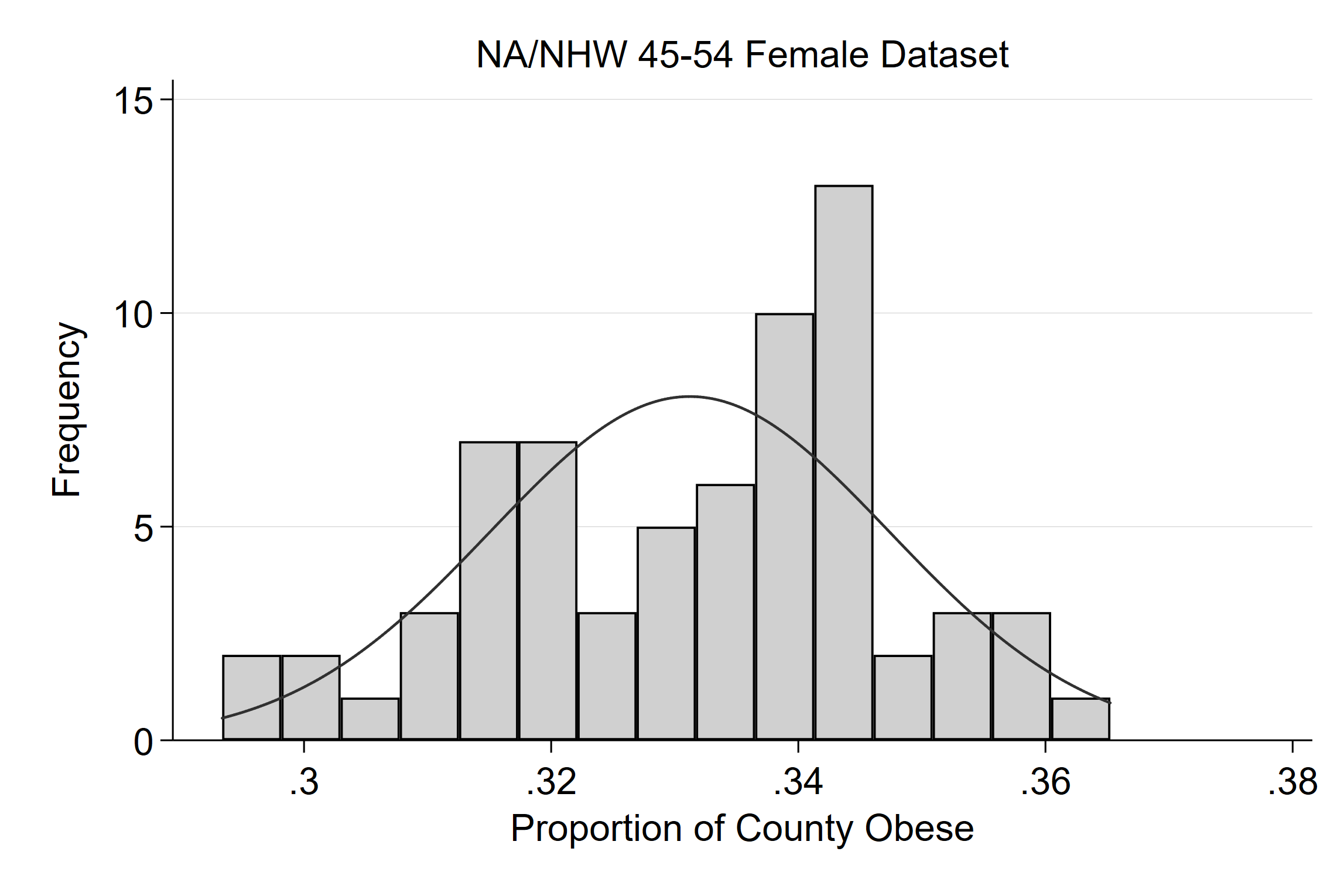

Supplement: Supplementary Figure 2a — Histogram—County Population Obesity–Female Dataset. [file Image_3.PNG]

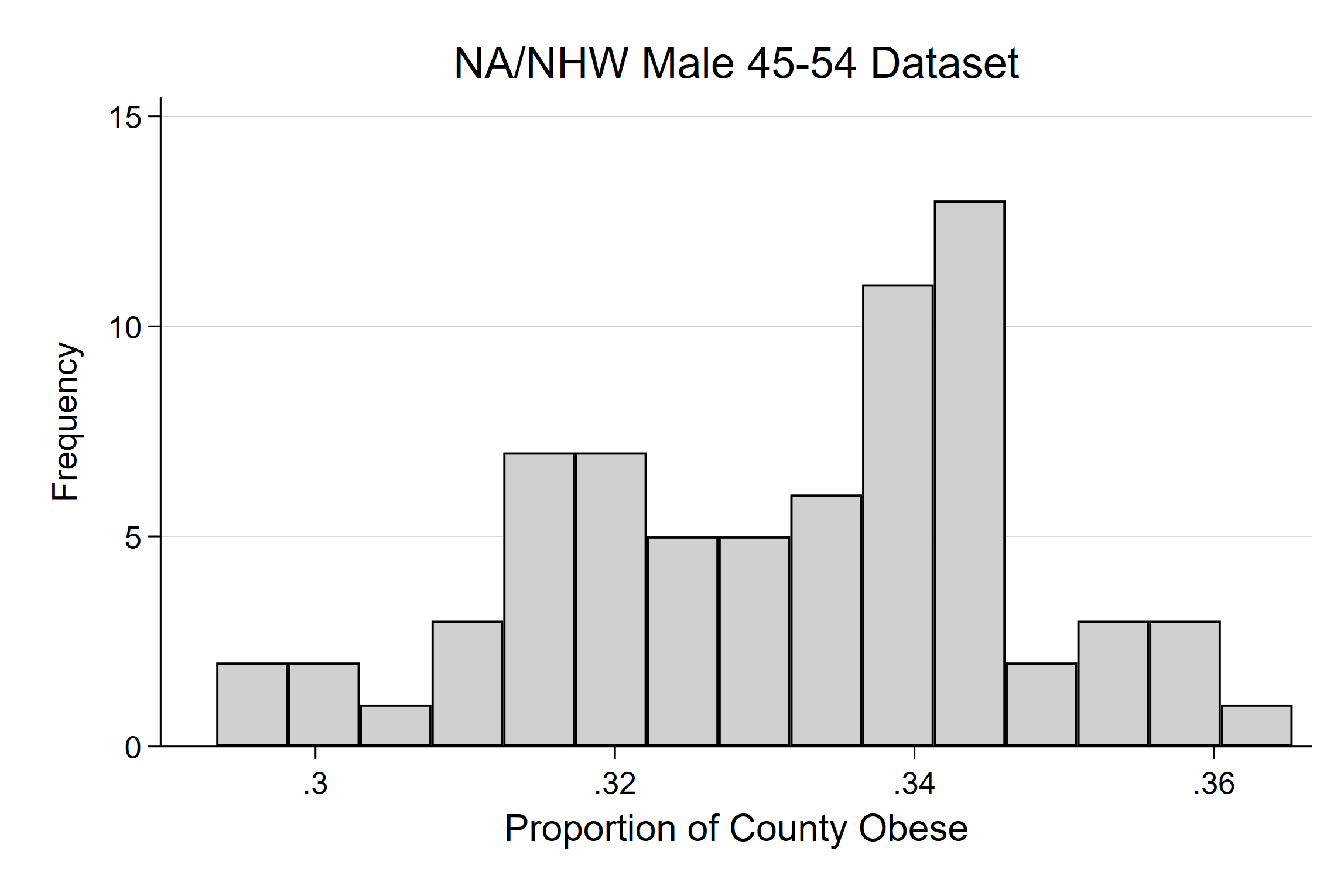

Supplement: Supplementary Figure 2b — Histogram—County Population Obesity–Male Dataset. [file Image_4.PNG]

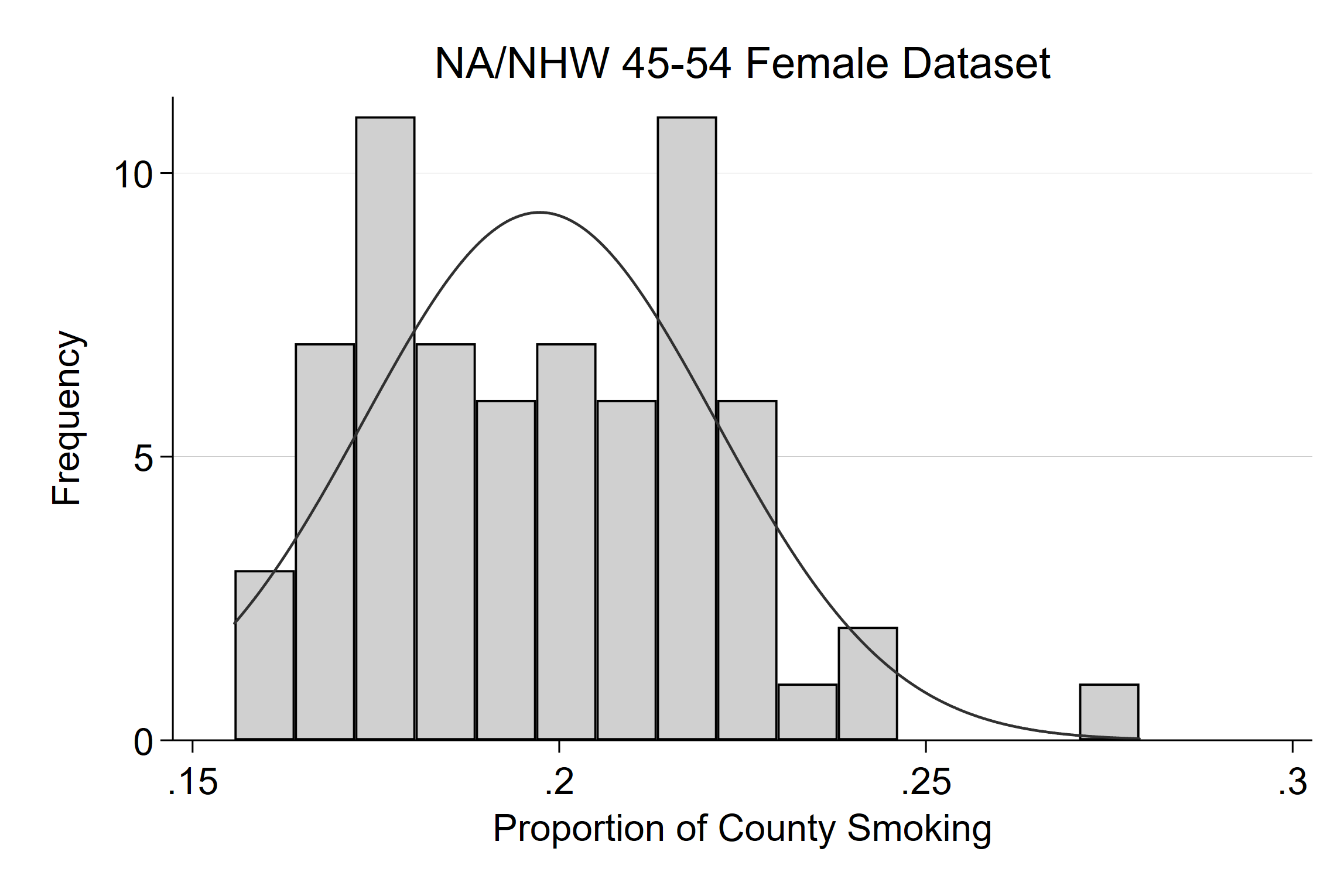

Supplement: Supplementary Figure 3a — County Smoking–Female Dataset. [file Image_5.PNG]

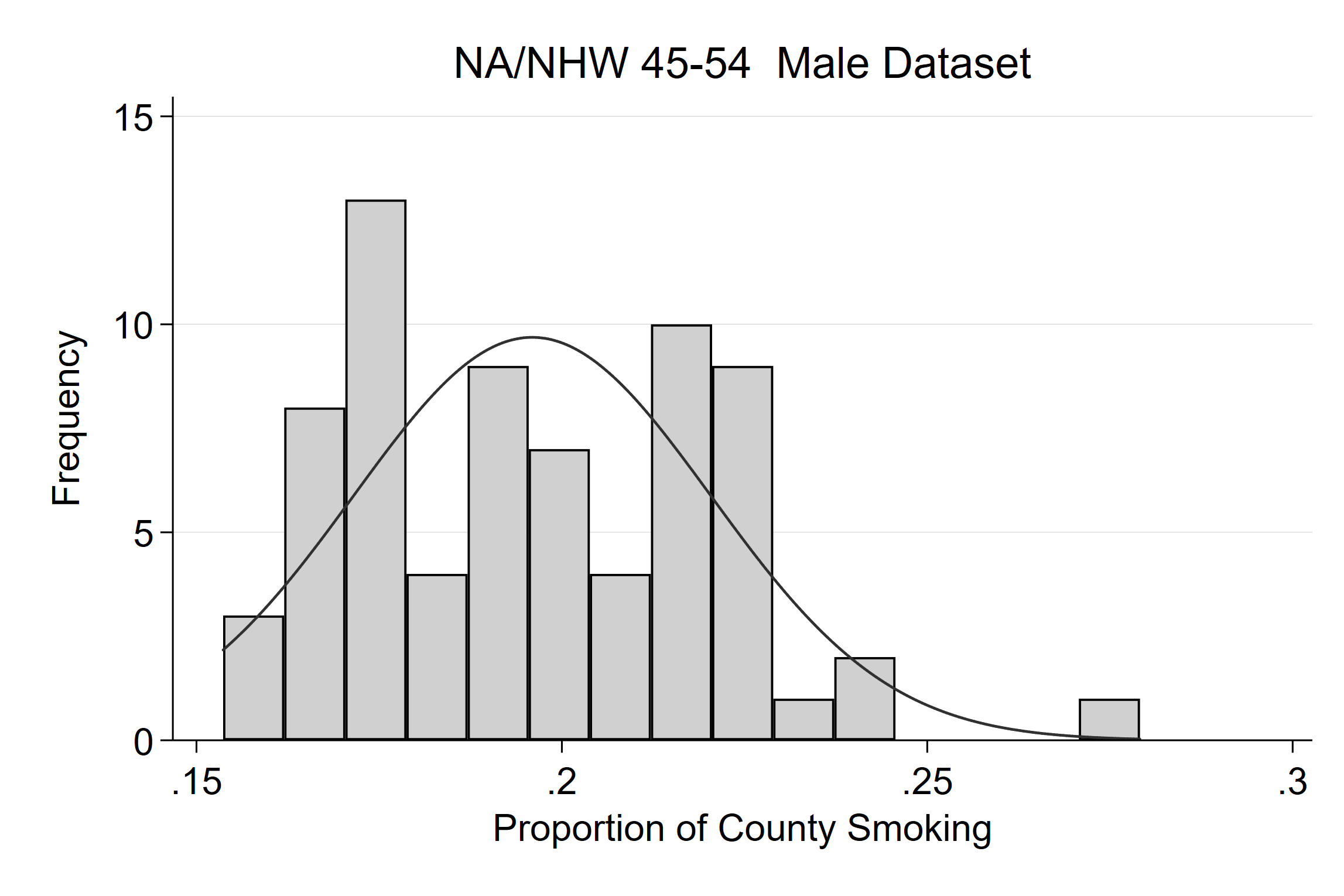

Supplement: Supplementary Figure 3b — County Smoking–Male Dataset. [file Image_6.PNG]

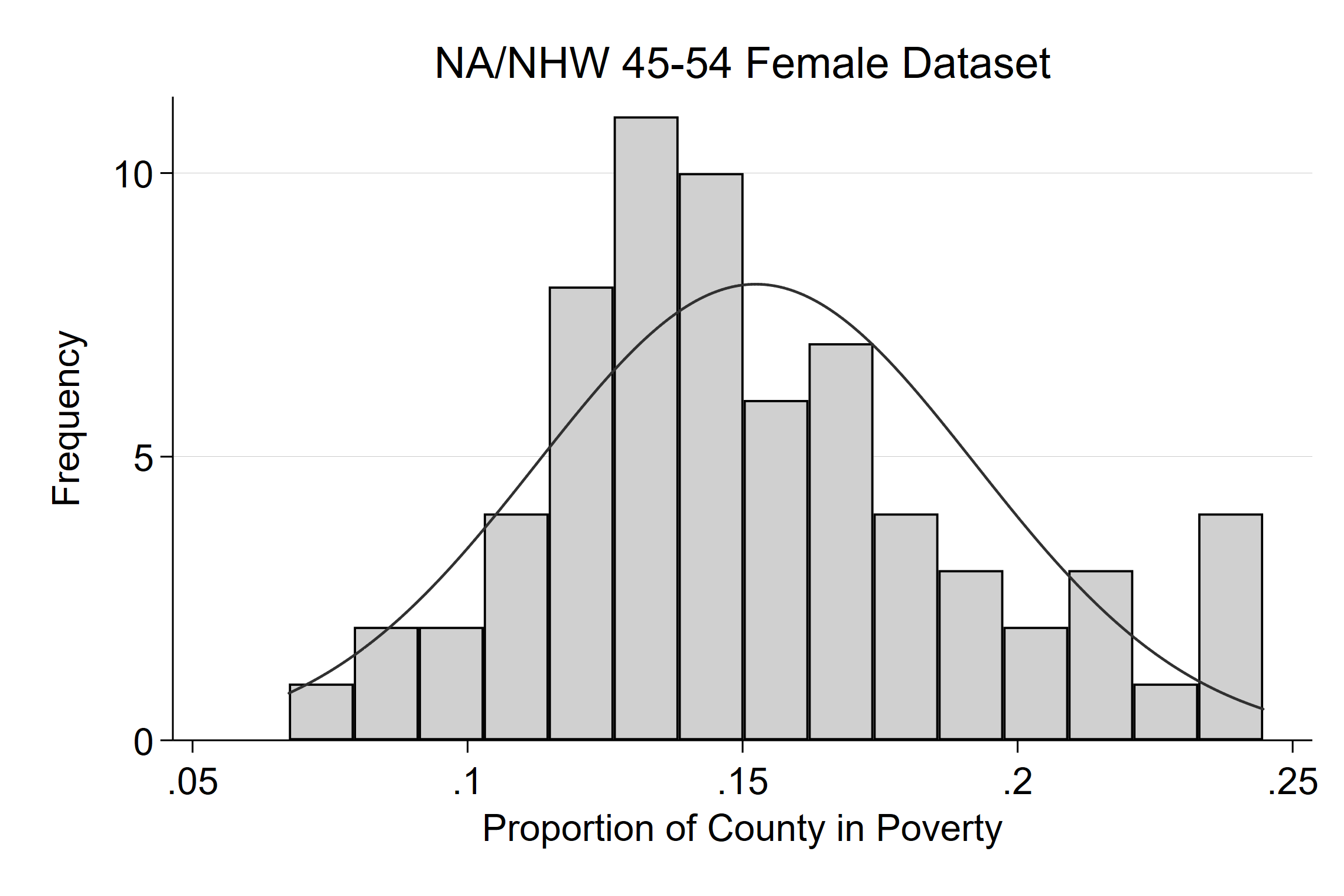

Supplement: Supplementary Figure 4a — Histogram—County Population in Poverty–Female Dataset. [file Image_7.PNG]

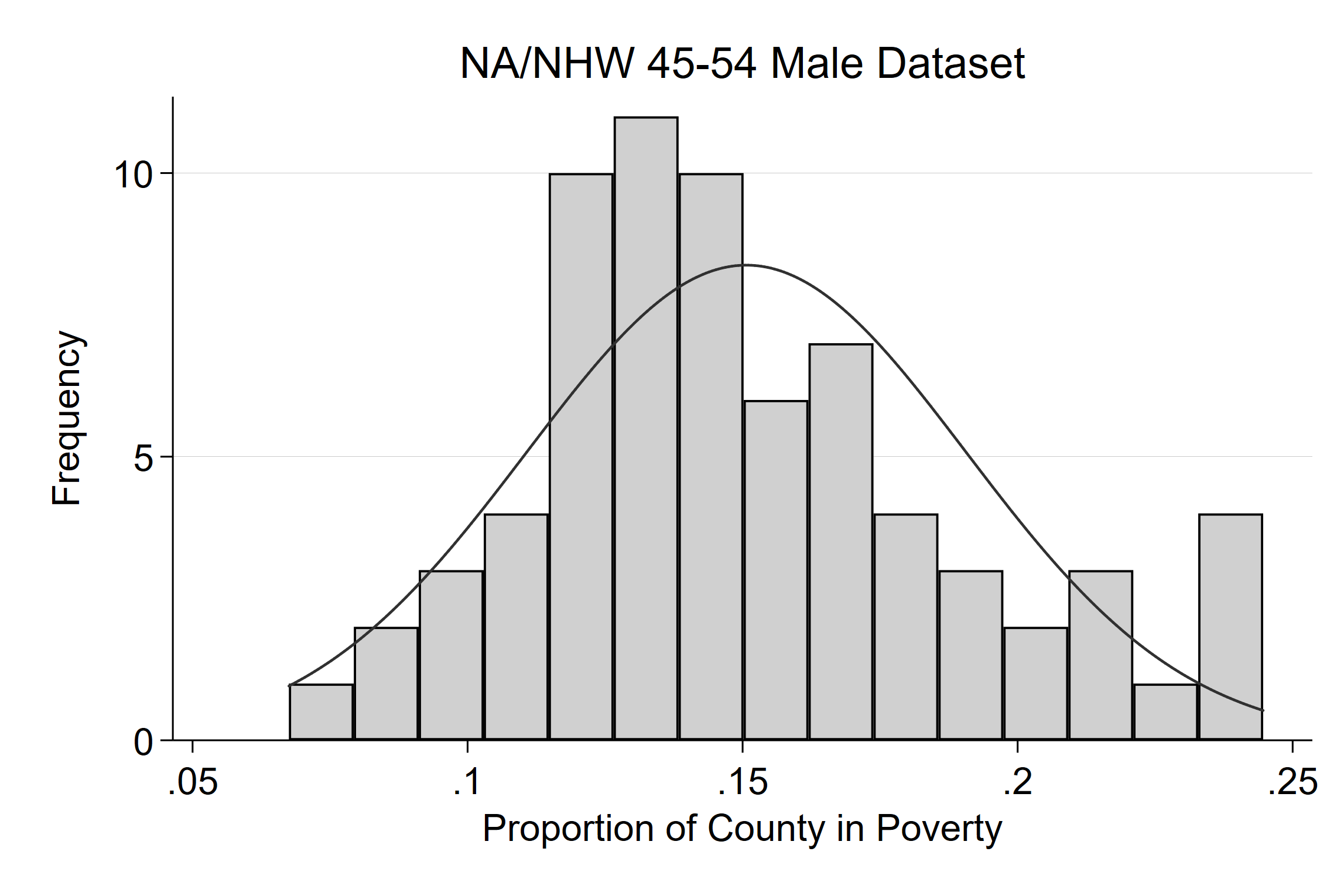

Supplement: Supplementary Figure 4b — Histogram—County Population in Poverty–Male Dataset. [file Image_8.PNG]

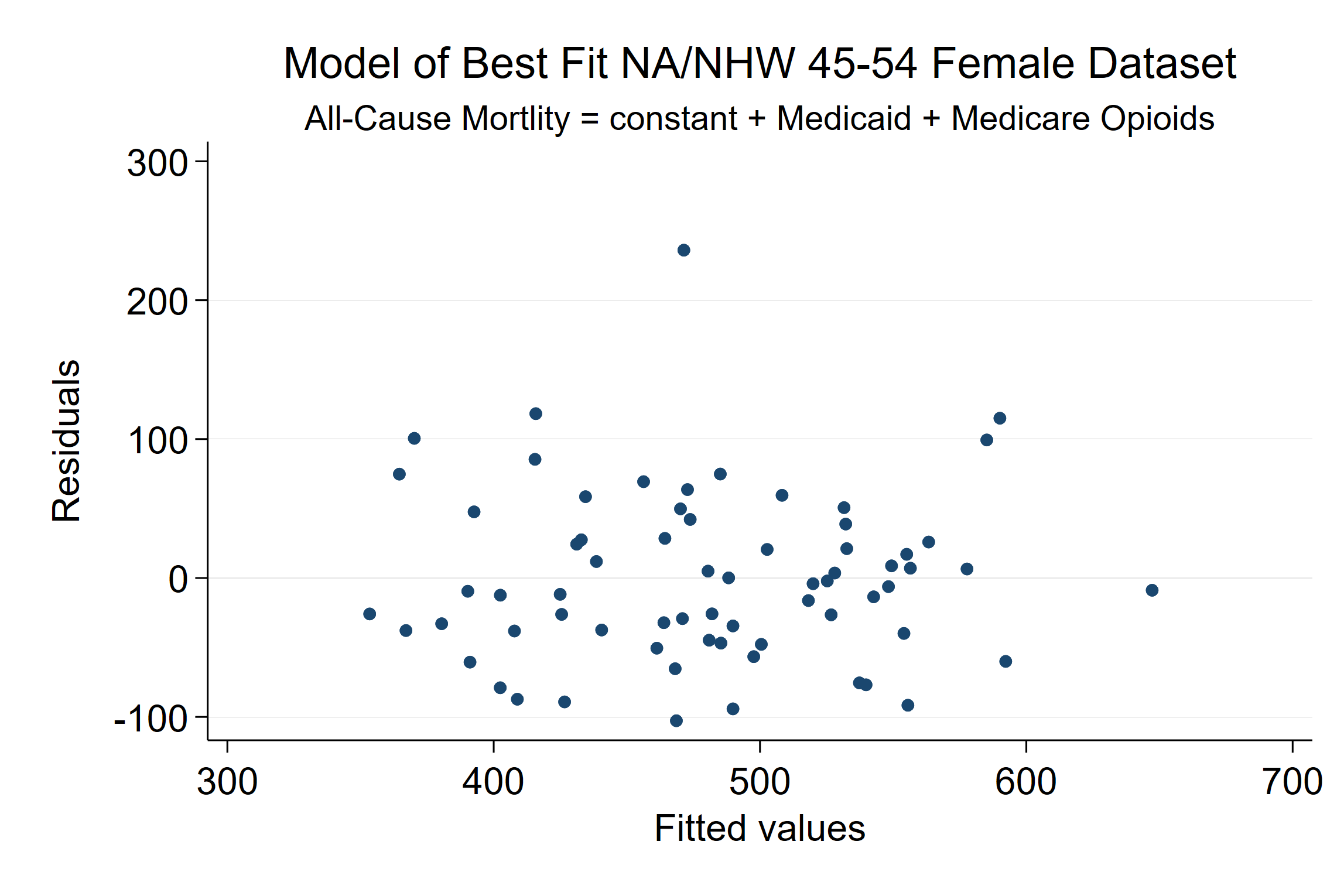

Supplement: Supplementary Figure 5a — Residuals vs. Fitted MAPC Medicaid vs. NHW45-54–Female Dataset. [file Image_9.PNG]

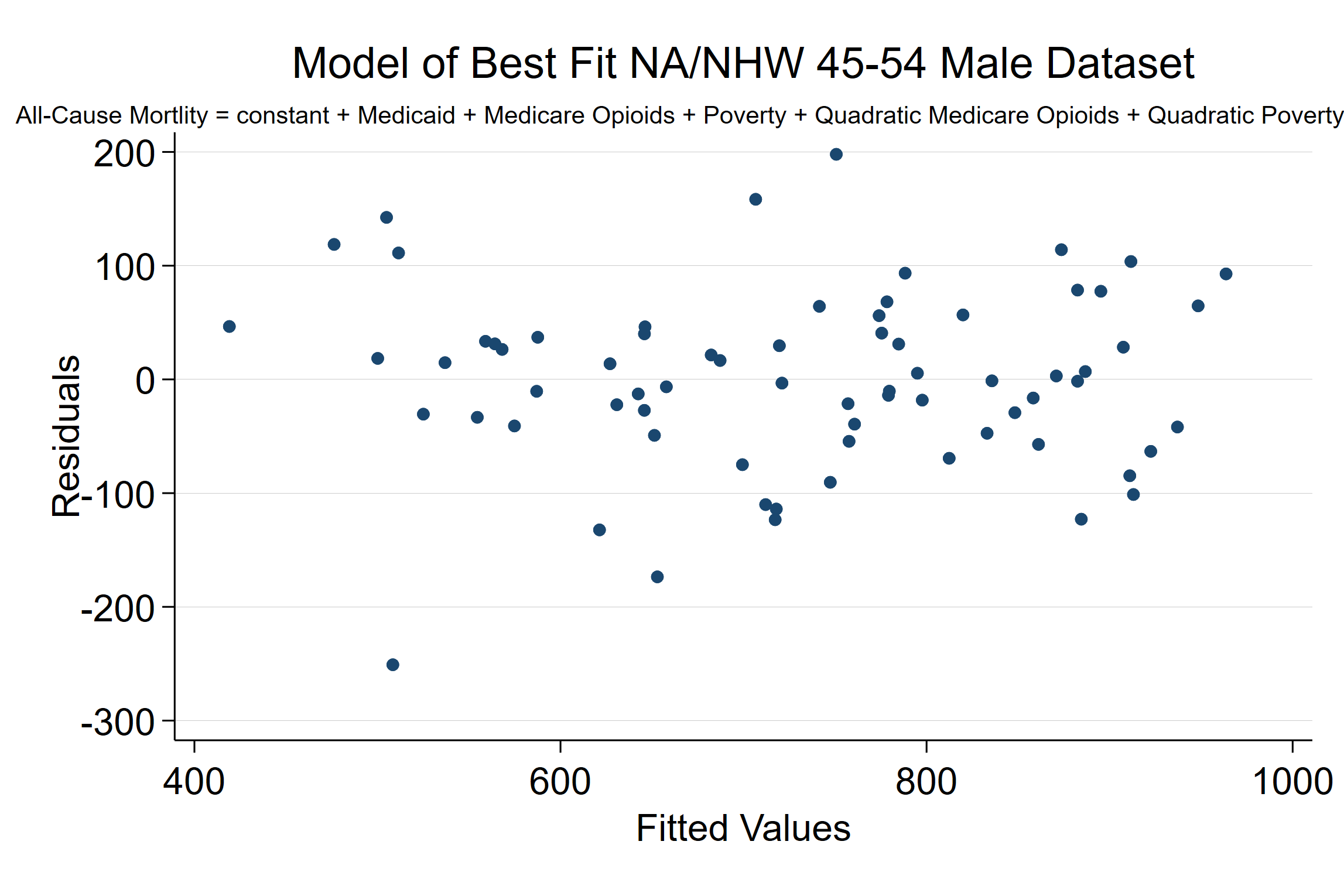

Supplement: Supplementary Figure 5b — Residuals vs. Fitted MAPC Medicaid vs. NHW45-54–Male Dataset. [file Image_10.PNG]
