# Supplementary material for: American Indian and Non-Hispanic White Midlife Mortality Is Associated With Medicaid Spending: An Oklahoma Ecological Study (1999–2016)
Source: Front Public Health. 2020 Apr 29;8:139. doi: 10.3389/fpubh.2020.00139 (PMC7202289; doi:10.3389/fpubh.2020.00139)
Supplement: Supplementary Table 1 — Online, publicly accessible databases used in this study. [file Table_1.DOCX]

| **Database** | **Variable** | **Years** | **Owner** |
| --- | --- | --- | --- |
| **CDC Wonder**  **Detailed Mortality**^[[1]](#endnote-1)^ | NA and NHW45-54 Mortality | (1999-2016) | US Government (CDC) |
| **Oklahoma Healthcare**  **Authority Annual Reports**^[[2]](#endnote-2)^ | Medicaid Spending | (1999-2016) | State of Oklahoma (OCHA) |
| **American FactFinder**^[[3]](#endnote-3)^ | Population & Poverty | (2005-2015) | US Census Bureau |
| **County Health Rankings & Roadmaps**^[[4]](#endnote-4)^ | Smoking | (2016-2018) | Robert Wood Johnson  Foundation |
| **County Health Rankings & Roadmaps**^[[5]](#endnote-5)^ | Obesity | (2010-2014) | Robert Wood Johnson  Foundation |

1. Centers for Disease Control and Prevention. About CDC Wonder. US Department of Health and Human Services June 19, 201 (https://wonder.cdc.gov/wonder/help/about-cdc-wonder-508.pdf) [↑](#endnote-ref-1)
2. Oklahoma Healthcare Authority. Archived Annual Reports. 2000-16. (http://www.okhca.org/research.aspx?id=9662&parts=7447) [↑](#endnote-ref-2)
3. U.S. Census Bureau. Download Center. American FactFinder. (<https://factfinder.census.gov/faces/nav/jsf/pages/download_center.xhtml>) [↑](#endnote-ref-3)
4. County Health Rankings & Roadmaps: Building a Culture of Health, County by County. Oklahoma Adult Smoking. Robert Wood Johnson Foundation. (http://www.countyhealthrankings.org/app/oklahoma/2018/measure/factors/9/map) [↑](#endnote-ref-4)
5. County Health Rankings & Roadmaps: Building a Culture of Health, County by County. Oklahoma Adult Obesity Robert Wood Johnson Foundation. (http://www.countyhealthrankings.org/app/oklahoma/2018/measure/factors/11/map) [↑](#endnote-ref-5)
