# Supplementary material for: American Indian and Non-Hispanic White Midlife Mortality Is Associated With Medicaid Spending: An Oklahoma Ecological Study (1999–2016)
Source: Front Public Health. 2020 Apr 29;8:139. doi: 10.3389/fpubh.2020.00139 (PMC7202289; doi:10.3389/fpubh.2020.00139)
Supplement: Supplementary Table 2a — Simple linear regressions between annual Oklahoma AI-NHW45-54 all-cause mortality and Medicaid spending per capita statewide (1999-16). [file Table_2.DOCX]

| **Gender** | **r** | **β** | **R2** | **SE** | **p** | **CI95** |
| --- | --- | --- | --- | --- | --- | --- |
| **Females** | 0.977 | 0.168 | 0.956 | 0.009 | < 0.001 | 0.149 0.187 |
| **Males** | 0.864 | 0.139 | 0.746 | 0.020 | < 0.001 | 0.096 0.182 |

**Table 2a. - Correlations between Annual Oklahoma NA-NHW45-54 All-Cause**

**Mortality and Medicaid Spending Per Capita Statewide and** **(1999-16)**

| **Gender** | **r** | **β** | **R2** | **SE** | **p** | **CI95** |
| --- | --- | --- | --- | --- | --- | --- |
| **Females** | 0.792 | 0.236 | 0.627 | 0.046 | < 0.001 | 0.140 0.333 |
| **Males** | 0.866 | 0.371 | 0.750 | .0535 | < 0.001 | 0.257 0.484 |

**Table 2b. - Correlations between Annual Oklahoma NA All-Cause**

**Mortality and Medicaid Spending Per Capita Statewide and (1999-16)**

| **Gender** | **r** | **β** | **R2** | **SE** | **p** | **CI95** |
| --- | --- | --- | --- | --- | --- | --- |
| **Females** | 0.968 | 0.155 | 0.937 | 0.010 | < 0.001 | 0.134, 0.177 |
| **Males** | 0.795 | 0.106 | 0.632 | 0.020 | < 0.001 | 0.063, 0.149 |

**Table 2c. - Correlations between Annual Oklahoma NHW45-54 All-Cause**

**Mortality and Medicaid Spending Per Capita Statewide and (1999-16)**
