# Supplementary material for: American Indian and Non-Hispanic White Midlife Mortality Is Associated With Medicaid Spending: An Oklahoma Ecological Study (1999–2016)
Source: Front Public Health. 2020 Apr 29;8:139. doi: 10.3389/fpubh.2020.00139 (PMC7202289; doi:10.3389/fpubh.2020.00139)
Supplement: Supplementary Table 3 — County-Level Variable Summaries. [file Table_3.DOCX]

**County-Level Variable Summaries**

|  | **Counties** | **Low** | **High** | **50%** | **Mean** | **Std. Dev.** | **Skewness** | **Kurtosis** |
| --- | --- | --- | --- | --- | --- | --- | --- | --- |
| **Female** | 66 | 316.00 | 821.00 | 479.65 | 481.83 | 93.74 | 0.79 | 4.48 |
| **Male** | 66 | 283.90 | 1029.00 | 745.95 | 732.3015 | 150.45 | -0.32 | 2.86 |

**Table 3a. Primary Outcome: NHW-45-54 All-Cause Mortality* (2000-16)**

*Deaths per 100 000 persons

| **Counties** | **Low** | **High** | **50%** | **Mean** | **Std.**  **Dev.** | **Skewness** | **Kurtosis** |
| --- | --- | --- | --- | --- | --- | --- | --- |
| 66 | 365.94 | 1535.11 | 934.06 | 920.36 | 284.16 | 0.09 | 2.09 |

**Table 3b. Primary Exposure: MAPC Medicaid Spending* (2000-16)**

*U.S. Dollars

| **Counties** | **Low** | **High** | **50%** | **Mean** | **Std.**  **Dev.** | **Skewness** | **Kurtosis** |
| --- | --- | --- | --- | --- | --- | --- | --- |
| 66 | 0.06 | 0.62 | 0.29 | 0.28 | 0.13 | 0.41 | 2.84 |

**Table 3c. Secondary Exposure: MAPC Medicare Opioid Claims (2013-14)**

| **Variable** | **Counties** | **Low** | **High** | **50%** | **Mean** | **Std. Dev.** | **Skewness** | **Kurtosis** |
| --- | --- | --- | --- | --- | --- | --- | --- | --- |
| **Smoking (2016-18)** | 66 | 0.16 | 0.28 | 0.20 | 0.20 | 0.02 | 0.57 | 3.40 |
| **Obesity**  **(2010-18)** | 66 | 0.29 | 0.37 | 0.33 | 0.33 | 0.016 | -0.28 | 2.42 |
| **Lack of High School Diploma**  **(2010-14)** | 66 | 0.03 | 0.42 | 0.19 | 0.19 | 0.06 | 0.29 | 4.98 |
| **Poverty**  **(2005-15)** | 66 | 0.068 | 0.245 | 0.147 | 0.153 | 0.040 | 0.441 | 2.859 |

**Table 3d. Covariate Exposure* Variables**

*Proportion of Individuals Surveyed
