# Supplementary material for: American Indian and Non-Hispanic White Midlife Mortality Is Associated With Medicaid Spending: An Oklahoma Ecological Study (1999–2016)
Source: Front Public Health. 2020 Apr 29;8:139. doi: 10.3389/fpubh.2020.00139 (PMC7202289; doi:10.3389/fpubh.2020.00139)
Supplement: Supplementary Table 4 — Correlations between mortality and explanatory variables. [file Table_4.DOCX]

|  | **Female**  **AI-NHW 45-54**  **Mortality** | **MAPC Medicaid Spending**  **(2000-2016)** | **MAPC Opioid Claims**  **(2013-2014)** |
| --- | --- | --- | --- |
| **MAPC Medicaid Spending (2000-2016)** | 0.696  p<0.001 | ------------ | 0.473  p<0.001 |
| **Mean Annual Medicare Opioid Claims (2013-2014)** | 0.513  p<0.001 | 0.473  p<0.001 | ------------- |
| **Co-variate Risk Factors** |  |  |  |
| **Smoking** | 0.601  p<0.001 | 0.756  p<0.001 | 0.390  p<0.001 |
| **Poverty** | 0.535  p<0.001 | 0.721  p<0.001 | 0.360  p<0.001 |
| **Obesity** | 0.418  p<0.001 | 0.505  p<0.001 | 0.120  p= 0.909 |

| **Co-variate Risk Factors** | **Smoking** | **Poverty** | **Obesity** |
| --- | --- | --- | --- |
| **Smoking** | 1.0 |  |  |
| **Poverty** | 0.812  p<0.001 | 1.0 |  |
| **Obesity** | 0.5574  p<0.001 | 0.376  p<0.001 | 1.0 |

**Table 4a.** **Pearson r correlations between county level AI-NHW45-54 mortality and explanatory variables (FEMALE mortality dataset)**

|  | **Male**  **AI-NHW 45-54**  **Mortality** | **MAPC Medicaid Spending**  **(2000-2016)** | **MAPC Opioid Claims**  **(2013-2014)** |
| --- | --- | --- | --- |
| **MAPC Medicaid Spending (2000-2016)** | 0.833  P<0.001 | ------------ | 0.495  P<0.001 |
| **Mean Annual Medicare Opioid Claims (2013-2014)** | 0.493  P<0.001 | 0.495  P<0.001 | ------------- |
| **Co-variate Risk Factors** |  |  |  |
| **Smoking** | 0.707  P<0.001 | 0.758  P<0.001 | 0.390  P<0.001 |
| **Obesity** | 0.471  P<0.001 | 0.501  P<0.001 | 0.120  P<0.001 |
| **Poverty** | 0.674  P<0.001 | 0.723  P<0.001 | 0.390  P<0.001 |

| **Co-variate Risk Factors** | **Smoking** | **Poverty** | **Obesity** |
| --- | --- | --- | --- |
| **Smoking** | 1.0 |  |  |
| **Obesity** | 0.538  P<0.001 | 0.368  P=0.002 | 1.0 |
| **Poverty** | 0.812  P<0.001 | 1.0 |  |

**Table 4b. Pearson r correlations between county level AI-NHW45-54 mortality and explanatory variables (MALE mortality dataset)**
